# Diazido­bis[4,4,5,5-tetra­methyl-2-(1,3-thia­zol-2-yl)-2-imidazoline-1-oxyl-3-oxide-κ^2^ *O*,*N*]manganese(II)

**DOI:** 10.1107/S1600536809000786

**Published:** 2009-01-14

**Authors:** Jiu Li Chang, Zhi Yong Gao, Kai Jiang

**Affiliations:** a College of Chemistry and Environmental Science, Henan Normal University, Xinxiang 453002, People’s Republic of China

## Abstract

In the crystal structure of the title compound, [Mn(N_3_)_2_(C_10_H_14_N_3_O_2_S)_2_], the Mn(II) atom exhibits a roughly octa­hedral coordination geometry. The Mn(II) atom lies on an inversion centre, thus the asymmetric unit comprises one half-mol­ecule. The metal center is six-coordinated by two azide anions and by two chelating 4,4,5,5-tetra­methyl-2-(1,3-thia­zol-2-yl)-2-imidazoline-1-oxyl-3-oxide nitronyl nitroxide radical ligands, leading to two six-membered chelate rings.

## Related literature

For the design and synthesis of mol­ecule-based magnetic materials, see: Aoki *et al.* (2003 ▶). For nitronyl nitroxide radicals, see: Minguet *et al.* (2000 ▶); Catala *et al.* (2005 ▶). For transition metal–radical complexes, see: Wang *et al.* (2005 ▶). For paramagnetic metal complexes of nitronyl nitroxide radicals, see: Li *et al.* (2002 ▶); Liu *et al.* (2001 ▶). For the synthesis, see: Ullman *et al.* (1970 ▶, 1972 ▶)
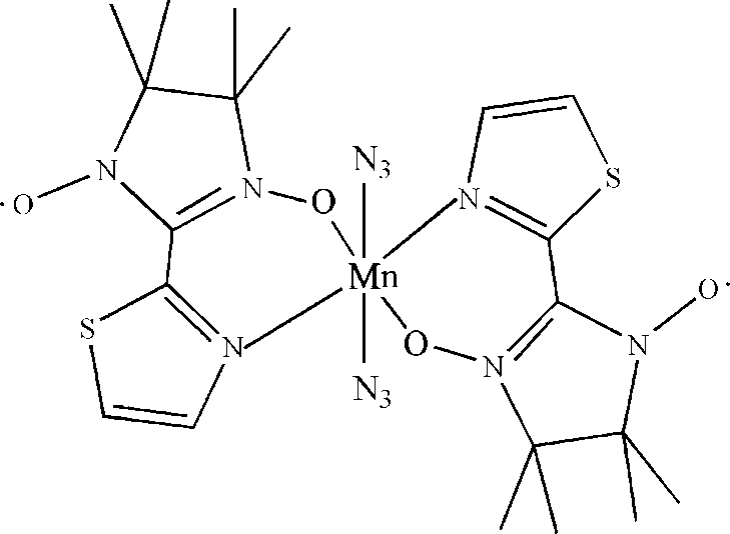


## Experimental

### 

#### Crystal data

[Mn(N_3_)_2_(C_10_H_14_N_3_O_2_S)_2_]
*M* *_r_* = 619.60
Monoclinic, 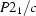

*a* = 9.9600 (18) Å
*b* = 12.272 (2) Å
*c* = 11.353 (2) Å
β = 103.714 (3)°
*V* = 1348.1 (4) Å^3^
*Z* = 2
Mo *K*α radiation
μ = 0.70 mm^−1^
*T* = 291 (2) K
0.45 × 0.30 × 0.25 mm

#### Data collection

Bruker SMART CCD area-detector diffractometer
Absorption correction: multi-scan (*SADABS*; Sheldrick, 1996 ▶) *T*_min_ = 0.745, *T*_max_ = 0.846
7966 measured reflections
3061 independent reflections
2628 reflections with *I* > 2σ(*I*)
*R*_int_ = 0.036

#### Refinement

*R*[*F* ^2^ > 2σ(*F* ^2^)] = 0.039
*wR*(*F* ^2^) = 0.110
*S* = 1.06
3061 reflections
182 parameters
H-atom parameters constrained
Δρ_max_ = 0.37 e Å^−3^
Δρ_min_ = −0.25 e Å^−3^

### 

Data collection: *SMART* (Bruker, 2002 ▶); cell refinement: *SAINT* (Bruker, 2002 ▶); data reduction: *SAINT*; program(s) used to solve structure: *SHELXS97* (Sheldrick, 2008 ▶); program(s) used to refine structure: *SHELXL97* (Sheldrick, 2008 ▶); molecular graphics: *SHELXTL* (Sheldrick, 2008 ▶); software used to prepare material for publication: *publCIF* (Westrip, 2009 ▶).

## Supplementary Material

Crystal structure: contains datablocks I, global. DOI: 10.1107/S1600536809000786/kp2202sup1.cif

Structure factors: contains datablocks I. DOI: 10.1107/S1600536809000786/kp2202Isup2.hkl

Additional supplementary materials:  crystallographic information; 3D view; checkCIF report

## Figures and Tables

**Fig. 1.**
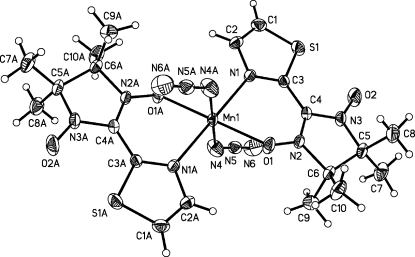
ORTEP drawing of the title compound with atom labeling. The thermal ellipsoids are drawn at 30% probability level.[symmetry codes: x,-y,-z + 1].

## supplementary crystallographic information

### Comment

The design and synthesis of molecule-based magnetic materials is one of the major subjects of materials science(Aoki *et al.* 2003). In many different types of organic radicals, research has focused on the nitronyl nitroxide radicals (NITR) family because of their flexibility and functionality (Minguet *et al.* 2000; Catala *et al.* 2005). The nitroxide derivatives can be bound to the metal center through the oxygen atoms of O–N groups, affording a good variety of transition metal–radical complexes (Wang *et al.* 2005;). There have been many magnetic studies on transition metal complexes with nitronyl nitroxide and imino nitroxide radicals and paramagnetic metal complexes of nitronyl nitroxide radicals have been extensively studied (Li *et al.* 2002; Liu *et al.* 2001). In the present paper, we report the synthesis and crystal structure of the title compound Mn(N_3_)_2_(NIT2-thz)_2_.

### Experimental

NIT2-thz [NIT2-thz = 4,4,5,5-tetramethyl-2-(1,3-thiazol-2-yl)-2-imidazoline-1-oxyl-3-oxide] was synthesized using a method in the literatrue (Ullman *et al.* 1970; Ullman *et al.* 1972). Mn (Ac)_2_. 4H_2_O(1 mmol) and NIT2-thz (2 mmol) were mixed in 30 ml of methanol. An aqueous solution (10 ml) of NaN_3_ (2 mmol) was added to this solution. The mixture was stirred for an 1 h and filtered off. The filtrate was kept at room temperatrue for 1 meek, and well formed dark brown crstals of Mn(N_3_)_2_(NIT2-thz)_2_ were obtained.

### Refinement

The H atoms were positioned geometrically and refined using the riding-model approximation, with C—H = 0.93 or 0.96Å and *U*_iso_(H) = 1.2Ueq(carrier) or *U*_iso_(H) = 1.5Ueq(methyl carrier).

### Crystal data

**Table:**

[Mn(N_3_)_2_(C_10_H_14_N_3_O_2_S)_2_]	*F*(000) = 642
*M**_r_* = 619.60	*D*_x_ = 1.526 Mg m^−^^3^
Monoclinic, *P*2_1_/*c*	Mo *K*α radiation, λ = 0.71073 Å
Hall symbol: -P 2ybc	Cell parameters from 4318 reflections
*a* = 9.9600 (18) Å	θ = 2.5–28.2°
*b* = 12.272 (2) Å	µ = 0.70 mm^−^^1^
*c* = 11.353 (2) Å	*T* = 291 K
β = 103.714 (3)°	Block, dark brown
*V* = 1348.1 (4) Å^3^	0.45 × 0.30 × 0.25 mm
*Z* = 2	

### Data collection

**Table:**

Bruker SMART CCD area-detector diffractometer	3061 independent reflections
Radiation source: fine-focus sealed tube	2628 reflections with *I* > 2σ(*I*)
graphite	*R*_int_ = 0.036
φ and ω scans	θ_max_ = 27.5°, θ_min_ = 2.5°
Absorption correction: multi-scan (*SADABS*; Sheldrick, 1996)	*h* = −12→12
*T*_min_ = 0.745, *T*_max_ = 0.846	*k* = −15→15
7966 measured reflections	*l* = −12→14

### Refinement

**Table:**

Refinement on *F*^2^	Primary atom site location: structure-invariant direct methods
Least-squares matrix: full	Secondary atom site location: difference Fourier map
*R*[*F*^2^ > 2σ(*F*^2^)] = 0.039	Hydrogen site location: inferred from neighbouring sites
*wR*(*F*^2^) = 0.110	H-atom parameters constrained
*S* = 1.06	*w* = 1/[σ^2^(*F*_o_^2^) + (0.0605*P*)^2^ + 0.2559*P*] where *P* = (*F*_o_^2^ + 2*F*_c_^2^)/3
3061 reflections	(Δ/σ)_max_ < 0.001
182 parameters	Δρ_max_ = 0.37 e Å^−^^3^
0 restraints	Δρ_min_ = −0.25 e Å^−^^3^

### Special details

**Table:**

Geometry. All e.s.d.'s (except the e.s.d. in the dihedral angle between two l.s. planes)are estimated using the full covariance matrix. The cell e.s.d.'s are takeninto account individually in the estimation of e.s.d.'s in distances, anglesand torsion angles; correlations between e.s.d.'s in cell parameters are onlyused when they are defined by crystal symmetry. An approximate (isotropic)treatment of cell e.s.d.'s is used for estimating e.s.d.'s involving l.s. planes.
Refinement. Refinement of *F*^2^ against ALL reflections. The weighted *R*-factor *wR* andgoodness of fit *S* are based on *F*^2^, conventional *R*-factors *R* are basedon *F*, with *F* set to zero for negative *F*^2^. The threshold expression of*F*^2^ > σ(*F*^2^) is used only for calculating *R*-factors(gt) *etc*. and isnot relevant to the choice of reflections for refinement. *R*-factors basedon *F*^2^ are statistically about twice as large as those based on *F*, and *R*-factors based on ALL data will be even larger.

### Fractional atomic coordinates and isotropic or equivalent isotropic displacement parameters (Å^2^)

**Table:**

	*x*	*y*	*z*	*U*_iso_*/*U*_eq_	
Mn1	0.0000	0.0000	0.5000	0.03345 (14)	
S1	0.25159 (5)	0.27889 (4)	0.36683 (5)	0.05049 (17)	
O1	0.17034 (15)	−0.08711 (11)	0.44955 (14)	0.0504 (4)	
O2	0.38630 (18)	0.14599 (13)	0.23568 (17)	0.0660 (5)	
N1	0.12384 (15)	0.14413 (11)	0.47278 (13)	0.0337 (3)	
N2	0.23912 (14)	−0.05108 (11)	0.37542 (13)	0.0336 (3)	
N3	0.34509 (15)	0.05765 (13)	0.27421 (14)	0.0407 (4)	
N4	−0.1080 (2)	0.00819 (18)	0.31193 (18)	0.0641 (6)	
N5	−0.08511 (17)	0.04681 (12)	0.22465 (15)	0.0433 (4)	
N6	−0.0640 (3)	0.08223 (18)	0.13715 (19)	0.0761 (7)	
C1	0.1569 (2)	0.32772 (15)	0.4616 (2)	0.0495 (5)	
H1	0.1479	0.4012	0.4782	0.059*	
C2	0.0969 (2)	0.24624 (14)	0.50955 (17)	0.0412 (4)	
H2	0.0413	0.2584	0.5635	0.049*	
C3	0.20718 (16)	0.14870 (12)	0.39806 (15)	0.0318 (3)	
C4	0.25886 (16)	0.05337 (13)	0.34979 (15)	0.0317 (3)	
C5	0.40415 (19)	−0.05207 (16)	0.25646 (16)	0.0406 (4)	
C6	0.30045 (18)	−0.12873 (14)	0.29998 (16)	0.0382 (4)	
C7	0.4089 (3)	−0.0651 (2)	0.12397 (19)	0.0628 (6)	
H7A	0.3179	−0.0551	0.0732	0.094*	
H7B	0.4417	−0.1367	0.1114	0.094*	
H7C	0.4702	−0.0116	0.1039	0.094*	
C8	0.5497 (2)	−0.0545 (2)	0.3366 (2)	0.0601 (6)	
H8A	0.6029	0.0037	0.3137	0.090*	
H8B	0.5923	−0.1230	0.3269	0.090*	
H8C	0.5461	−0.0455	0.4197	0.090*	
C9	0.1798 (2)	−0.1673 (2)	0.1994 (2)	0.0595 (6)	
H9A	0.1102	−0.1990	0.2346	0.089*	
H9B	0.2118	−0.2208	0.1507	0.089*	
H9C	0.1413	−0.1064	0.1496	0.089*	
C10	0.3643 (3)	−0.22455 (19)	0.3780 (2)	0.0620 (6)	
H10A	0.4243	−0.1981	0.4515	0.093*	
H10B	0.4167	−0.2680	0.3345	0.093*	
H10C	0.2925	−0.2682	0.3974	0.093*	

### Atomic displacement parameters (Å^2^)

**Table:**

	*U*^11^	*U*^22^	*U*^33^	*U*^12^	*U*^13^	*U*^23^
Mn1	0.0362 (2)	0.0333 (2)	0.0373 (2)	−0.00148 (13)	0.02166 (16)	0.00206 (13)
S1	0.0483 (3)	0.0342 (2)	0.0743 (4)	−0.00716 (19)	0.0251 (3)	0.0074 (2)
O1	0.0597 (9)	0.0351 (6)	0.0717 (10)	0.0065 (6)	0.0459 (8)	0.0095 (6)
O2	0.0697 (10)	0.0592 (9)	0.0861 (12)	−0.0037 (8)	0.0525 (10)	0.0169 (8)
N1	0.0365 (7)	0.0299 (7)	0.0381 (7)	0.0019 (5)	0.0156 (6)	−0.0003 (5)
N2	0.0325 (7)	0.0336 (7)	0.0395 (8)	0.0037 (5)	0.0182 (6)	0.0003 (6)
N3	0.0371 (8)	0.0466 (8)	0.0452 (8)	0.0000 (6)	0.0230 (7)	0.0038 (7)
N4	0.0681 (13)	0.0827 (14)	0.0416 (10)	−0.0251 (10)	0.0134 (9)	0.0060 (9)
N5	0.0497 (9)	0.0360 (8)	0.0453 (9)	−0.0042 (6)	0.0138 (7)	0.0000 (7)
N6	0.121 (2)	0.0587 (12)	0.0582 (12)	−0.0128 (12)	0.0400 (13)	0.0104 (10)
C1	0.0490 (11)	0.0305 (8)	0.0668 (13)	0.0003 (8)	0.0093 (10)	−0.0061 (8)
C2	0.0449 (10)	0.0352 (8)	0.0444 (10)	0.0065 (7)	0.0122 (8)	−0.0065 (7)
C3	0.0297 (8)	0.0302 (7)	0.0372 (8)	−0.0012 (6)	0.0113 (6)	0.0028 (6)
C4	0.0277 (8)	0.0361 (8)	0.0335 (8)	0.0006 (6)	0.0118 (6)	0.0029 (6)
C5	0.0346 (9)	0.0556 (11)	0.0361 (9)	0.0076 (7)	0.0170 (7)	−0.0030 (8)
C6	0.0369 (9)	0.0404 (9)	0.0402 (9)	0.0095 (7)	0.0151 (7)	−0.0048 (7)
C7	0.0640 (14)	0.0898 (17)	0.0422 (11)	0.0087 (12)	0.0277 (10)	−0.0064 (11)
C8	0.0358 (10)	0.0856 (16)	0.0599 (13)	0.0054 (10)	0.0133 (10)	−0.0127 (12)
C9	0.0569 (13)	0.0618 (13)	0.0587 (13)	−0.0084 (10)	0.0117 (11)	−0.0173 (10)
C10	0.0695 (15)	0.0542 (12)	0.0703 (15)	0.0292 (11)	0.0324 (13)	0.0111 (11)

### Geometric parameters (Å, °)

**Table:**

Mn1—N4^i^	2.153 (2)	C2—H2	0.9300
Mn1—N4	2.153 (2)	C3—C4	1.438 (2)
Mn1—O1^i^	2.1931 (12)	C5—C8	1.518 (3)
Mn1—O1	2.1931 (12)	C5—C7	1.525 (3)
Mn1—N1	2.2194 (14)	C5—C6	1.561 (3)
Mn1—N1^i^	2.2194 (14)	C6—C10	1.518 (3)
S1—C1	1.699 (2)	C6—C9	1.524 (3)
S1—C3	1.7170 (16)	C7—H7A	0.9600
O1—N2	1.2832 (17)	C7—H7B	0.9600
O2—N3	1.273 (2)	C7—H7C	0.9600
N1—C3	1.321 (2)	C8—H8A	0.9600
N1—C2	1.367 (2)	C8—H8B	0.9600
N2—C4	1.339 (2)	C8—H8C	0.9600
N2—C6	1.504 (2)	C9—H9A	0.9600
N3—C4	1.3513 (19)	C9—H9B	0.9600
N3—C5	1.502 (2)	C9—H9C	0.9600
N4—N5	1.168 (2)	C10—H10A	0.9600
N5—N6	1.148 (2)	C10—H10B	0.9600
C1—C2	1.345 (3)	C10—H10C	0.9600
C1—H1	0.9300		
			
N4^i^—Mn1—N4	180.0	N2—C4—C3	127.70 (14)
N4^i^—Mn1—O1^i^	89.95 (8)	N3—C4—C3	123.31 (15)
N4—Mn1—O1^i^	90.05 (8)	N3—C5—C8	106.60 (17)
N4^i^—Mn1—O1	90.05 (8)	N3—C5—C7	109.34 (17)
N4—Mn1—O1	89.95 (8)	C8—C5—C7	109.88 (17)
O1^i^—Mn1—O1	180.0	N3—C5—C6	100.84 (13)
N4^i^—Mn1—N1	90.68 (6)	C8—C5—C6	114.10 (17)
N4—Mn1—N1	89.32 (6)	C7—C5—C6	115.27 (18)
O1^i^—Mn1—N1	97.92 (5)	N2—C6—C10	109.20 (15)
O1—Mn1—N1	82.08 (5)	N2—C6—C9	105.59 (15)
N4^i^—Mn1—N1^i^	89.32 (6)	C10—C6—C9	110.10 (19)
N4—Mn1—N1^i^	90.68 (6)	N2—C6—C5	100.77 (13)
O1^i^—Mn1—N1^i^	82.08 (5)	C10—C6—C5	115.73 (16)
O1—Mn1—N1^i^	97.92 (5)	C9—C6—C5	114.43 (16)
N1—Mn1—N1^i^	180.0	C5—C7—H7A	109.5
C1—S1—C3	89.35 (9)	C5—C7—H7B	109.5
N2—O1—Mn1	124.73 (10)	H7A—C7—H7B	109.5
C3—N1—C2	110.83 (14)	C5—C7—H7C	109.5
C3—N1—Mn1	125.29 (11)	H7A—C7—H7C	109.5
C2—N1—Mn1	122.15 (11)	H7B—C7—H7C	109.5
O1—N2—C4	126.95 (13)	C5—C8—H8A	109.5
O1—N2—C6	120.47 (13)	C5—C8—H8B	109.5
C4—N2—C6	112.47 (13)	H8A—C8—H8B	109.5
O2—N3—C4	123.82 (15)	C5—C8—H8C	109.5
O2—N3—C5	123.29 (14)	H8A—C8—H8C	109.5
C4—N3—C5	112.24 (14)	H8B—C8—H8C	109.5
N5—N4—Mn1	135.07 (17)	C6—C9—H9A	109.5
N6—N5—N4	178.1 (2)	C6—C9—H9B	109.5
C2—C1—S1	111.16 (14)	H9A—C9—H9B	109.5
C2—C1—H1	124.4	C6—C9—H9C	109.5
S1—C1—H1	124.4	H9A—C9—H9C	109.5
C1—C2—N1	114.81 (16)	H9B—C9—H9C	109.5
C1—C2—H2	122.6	C6—C10—H10A	109.5
N1—C2—H2	122.6	C6—C10—H10B	109.5
N1—C3—C4	123.11 (14)	H10A—C10—H10B	109.5
N1—C3—S1	113.83 (12)	C6—C10—H10C	109.5
C4—C3—S1	123.04 (12)	H10A—C10—H10C	109.5
N2—C4—N3	108.87 (14)	H10B—C10—H10C	109.5
			
N4^i^—Mn1—O1—N2	−122.97 (15)	O1—N2—C4—N3	175.59 (17)
N4—Mn1—O1—N2	57.03 (15)	C6—N2—C4—N3	−8.19 (19)
O1^i^—Mn1—O1—N2	105 (8)	O1—N2—C4—C3	−0.6 (3)
N1—Mn1—O1—N2	−32.28 (14)	C6—N2—C4—C3	175.65 (17)
N1^i^—Mn1—O1—N2	147.72 (14)	O2—N3—C4—N2	−178.27 (17)
N4^i^—Mn1—N1—C3	118.52 (15)	C5—N3—C4—N2	−7.24 (19)
N4—Mn1—N1—C3	−61.48 (15)	O2—N3—C4—C3	−1.9 (3)
O1^i^—Mn1—N1—C3	−151.43 (14)	C5—N3—C4—C3	169.12 (16)
O1—Mn1—N1—C3	28.57 (14)	N1—C3—C4—N2	−3.7 (3)
N1^i^—Mn1—N1—C3	24.5 (17)	S1—C3—C4—N2	174.59 (14)
N4^i^—Mn1—N1—C2	−77.84 (15)	N1—C3—C4—N3	−179.39 (16)
N4—Mn1—N1—C2	102.16 (15)	S1—C3—C4—N3	−1.1 (2)
O1^i^—Mn1—N1—C2	12.20 (15)	O2—N3—C5—C8	70.0 (2)
O1—Mn1—N1—C2	−167.80 (15)	C4—N3—C5—C8	−101.13 (18)
N1^i^—Mn1—N1—C2	−171.9 (18)	O2—N3—C5—C7	−48.8 (2)
Mn1—O1—N2—C4	26.1 (2)	C4—N3—C5—C7	140.13 (18)
Mn1—O1—N2—C6	−149.81 (13)	O2—N3—C5—C6	−170.64 (18)
N4^i^—Mn1—N4—N5	−71 (2)	C4—N3—C5—C6	18.27 (18)
O1^i^—Mn1—N4—N5	118.9 (3)	O1—N2—C6—C10	−42.4 (2)
O1—Mn1—N4—N5	−61.1 (3)	C4—N2—C6—C10	141.14 (17)
N1—Mn1—N4—N5	21.0 (3)	O1—N2—C6—C9	76.0 (2)
N1^i^—Mn1—N4—N5	−159.0 (3)	C4—N2—C6—C9	−100.50 (18)
Mn1—N4—N5—N6	137 (8)	O1—N2—C6—C5	−164.65 (15)
C3—S1—C1—C2	−0.76 (16)	C4—N2—C6—C5	18.85 (18)
S1—C1—C2—N1	−0.1 (2)	N3—C5—C6—N2	−20.40 (16)
C3—N1—C2—C1	1.2 (2)	C8—C5—C6—N2	93.45 (18)
Mn1—N1—C2—C1	−164.58 (14)	C7—C5—C6—N2	−138.01 (17)
C2—N1—C3—C4	176.71 (16)	N3—C5—C6—C10	−138.01 (17)
Mn1—N1—C3—C4	−18.1 (2)	C8—C5—C6—C10	−24.2 (2)
C2—N1—C3—S1	−1.76 (19)	C7—C5—C6—C10	104.4 (2)
Mn1—N1—C3—S1	163.46 (8)	N3—C5—C6—C9	92.35 (18)
C1—S1—C3—N1	1.47 (15)	C8—C5—C6—C9	−153.79 (17)
C1—S1—C3—C4	−177.00 (16)	C7—C5—C6—C9	−25.3 (2)

Symmetry codes: (i) −*x*, −*y*, −*z*+1.
